# Glomus Tumor of the Chest Wall With Metastases to Lung

**DOI:** 10.7759/cureus.69122

**Published:** 2024-09-10

**Authors:** Joshua A Engle, James T Dibb, Soumajit Kundu, John A Jakob

**Affiliations:** 1 Department of Medicine, Summa Health, Akron, USA; 2 Department of Medical Oncology, Summa Health, Akron, USA

**Keywords:** dermatology, dermato-pathology, glomus tumor, lung metastasis, neuroendocrine tumor, oncology, soft tissue neoplasm

## Abstract

Glomus tumors are typically benign, soft tissue neoplasms composed of thermoregulatory glomus bodies. The more common varieties, such as subungual, are treated surgically and typically have a very low mortality rate. Malignant glomus tumors are very rare, and their pathogenesis is poorly understood. As such, treatment options and prognosis are unclear.

We present a 67-year-old female diagnosed with a chest wall glomus tumor with biopsy-proven metastases to her lungs. Her treatment course included neoadjuvant radiation therapy followed by immunotherapy with pembrolizumab. After completion of the initial radiation therapy, imaging showed disease regression. Interval imaging after seven months of immunotherapy showed the resolution of all lung nodules with no reported concerns for disease recurrence. Pembrolizumab was discontinued due to concerns for dermatologic and renal adverse events, and the patient continues to be monitored off therapy.

The metastatic glomus tumor described in this case had several unique qualities, including its initial presentation on the chest wall as an aggressive lesion, as well as its spread to multiple locations in the lungs. Glomus tumors are not normally as aggressive as seen in this case, but the genetic profile with high tumor mutational burden allowed for guided treatment. Radiation is often used as neoadjuvant treatment in higher risk glomus tumors, but the addition of immunotherapy such as pembrolizumab represents a potential avenue to manage these patients when surgery is not an option.

Malignant glomus tumors are exceptionally rare occurrences that, by nature of their rarity, require protocols or therapies that are not specifically designed for their treatment. The clinical course of these tumors is difficult to predict as most cases of metastatic spread have few examples from which to draw conclusions. This case provided encouraging results for treatment with radiation and, potentially, immunotherapy. Each instance of a malignant glomus tumor and its genetic profile should be closely examined and documented so that sufficient data can be accumulated to guide treatment for this rare cancer.

## Introduction

Glomus tumors are typically benign mesenchymal neoplasms that make up less than 2% of soft tissue tumors. They are composed of glomus bodies, which are specialized arteriovenous anastomoses in the dermis responsible for thermoregulation. Glomus tumors most commonly arise in the subungual regions of the digits or in the deep dermis of the upper and lower extremities, though they have been described to manifest in several other locations where glomus bodies do not characteristically occur, including the mediastinum, bone, penis, and internal organs such as the stomach, small bowel, and lung. A subungual manifestation is more common in women, though there is no clear sex predisposition seen for other sites of disease [1].

Malignant glomus tumors, also known as glomangiosarcomas, are exceptionally rare [2]. The development of malignant potential is poorly understood, but in 2001, Folpe et al. proposed a novel classification of atypical or malignant glomus tumors, categorized as being malignant, symplastic, a glomus tumor of uncertain malignant potential, or a glomangiomatosis. This classification system would form the basis for the current WHO diagnostic criterion for these tumors. Malignant glomus tumors are now characterized by atypical mitotic figures, indicating atypical cellular division or marked nuclear atypia, meaning abnormal nuclei, regardless of mitotic activity. Tumors of uncertain malignant potential are characterized by not meeting the criteria for malignancy but having at least one atypical feature other than nuclear pleomorphism [3]. Approximately 3% of glomus tumors become malignant [2]. Metastatic glomus tumors are even rarer, occurring in less than 1% of cases. Incidences of metastatic spread vary widely and depend on the original location of the tumor at diagnosis [4]. The paucity of available cases, therefore, renders the duration of treatment and prediction of prognosis challenging.

Treatment options for glomus tumors vary. Typical cases are often amenable to surgical resection. Malignant examples are often treated with surgical resection in addition to chemotherapy. When these tumors arise in areas that are not amenable to resection, such as the bladder, radiation therapy has been utilized with some success [5]. These tumors remain so uncommon and variable in their presentations that no guideline recommendations or expert consensus exists regarding treatment beyond surgical excision [6]. As such, tumor location and genetic characterization are often used to help guide treatment based on the management of other types of cancer [5]. The unclear nature of treatment options correlates with the difficulty of determining prognosis as well. The more common subungual cases often have very low mortality rates. Malignant cases, especially those that metastasize, often portend very poor prognoses [7]. One study determined that 38% (eight of 21 patients) of patients whose glomus tumors met the criteria for malignancy metastasized. Of those patients with evidence of metastasis, six out of the eight had died from their disease after three years [6].

With this in mind, we report a case of malignant glomus tumor with initial presentation on the anterior chest wall as subcutaneous nodules with biopsy-proven metastases to the lung that were responsive to non-surgical treatment.

## Case presentation

**Figure 1:**
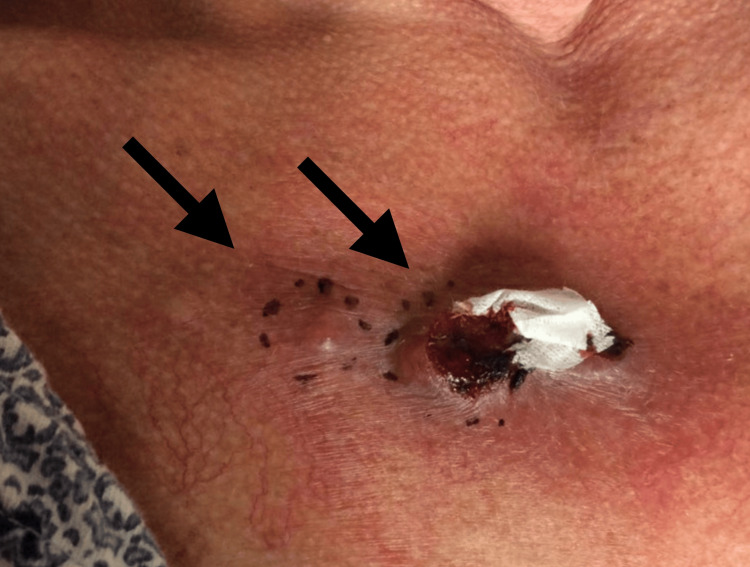
Anterior chest wall lesions diagnosed as glomus tumors following shave biopsy, characterized as subcutaneous nodules that were friable on presentation

**Figure 2:**
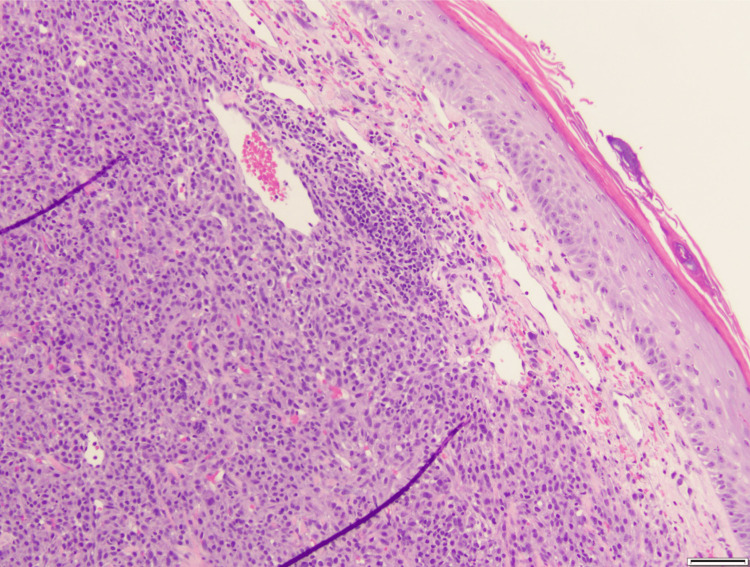
Shave biopsy of patient’s anterior chest wall lesions Malignant glomus tumor. Positive staining for smooth muscle actin and epithelial membrane antigen. Negative: S100, SRY Box (SOX)-10, melanoma antigen recognized by T cells (MART)-1, human melanoma black (HMB)-45, cytokeratin 5/6, P63, anti-cytokeratin (CAM) 5.2, pan-cytokeratin, erythroblast transformation specific-related gene (ERG), cluster of differentiation (CD) 68, 45, and 34, and desmin.

**Figure 3:**
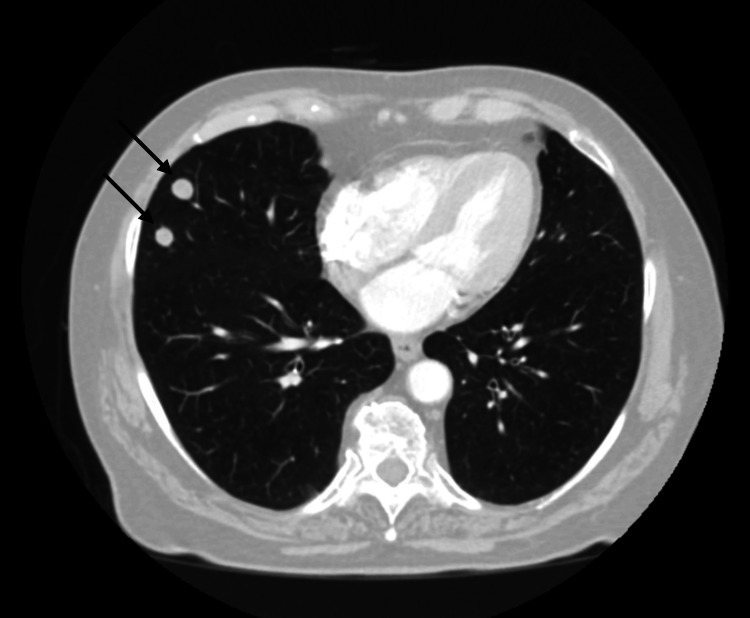
Initial CT of the chest, abdomen, and pelvis, which showed two right middle lobe nodules, concerning for the metastatic process

**Figure 4:**
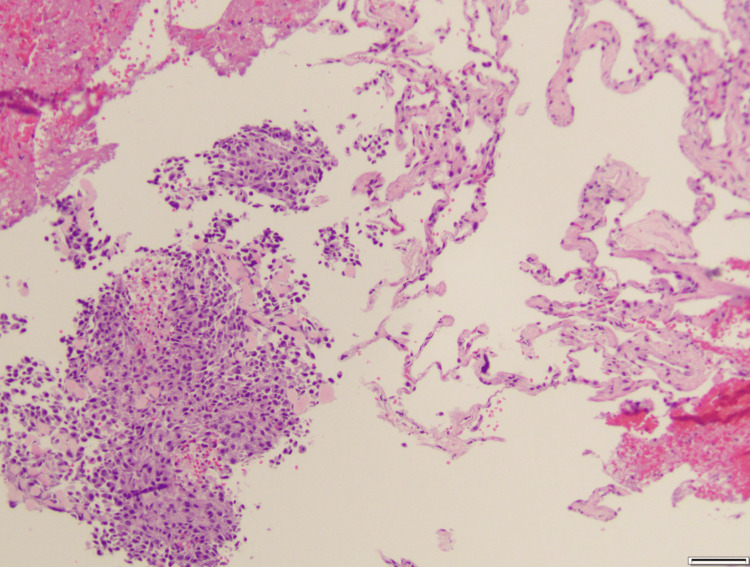
CT-guided needle core biopsy of right middle lung nodule Positive staining for smooth muscle actin and epithelial membrane antigen. Negative: transcription termination factor (TTF)-1 and pan-cytokeratin. This pattern indicated involvement with the patient’s primary glomus tumor.

**Figure 5:**
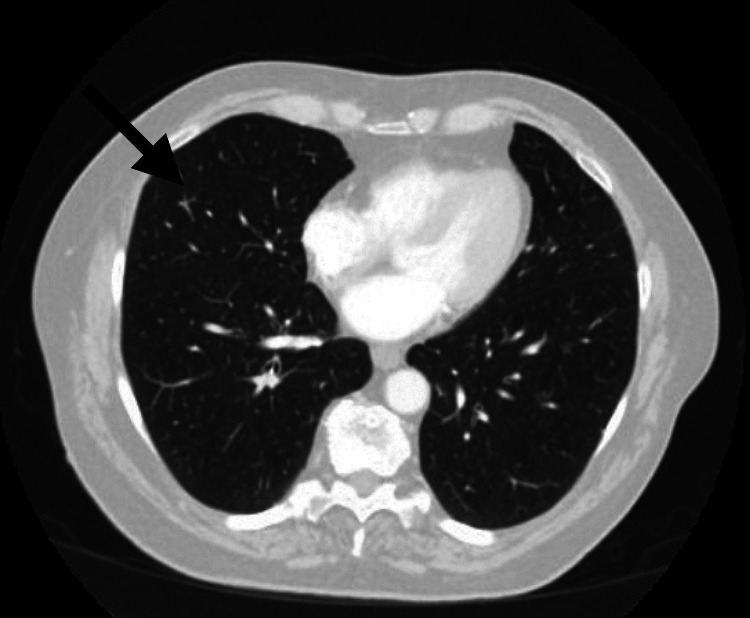
CT of the chest showing evidence of resolving right middle lobe nodules (previously seen in the location indicated with an arrow) after radiation to the chest wall and before pembrolizumab therapy

**Figure 6:**
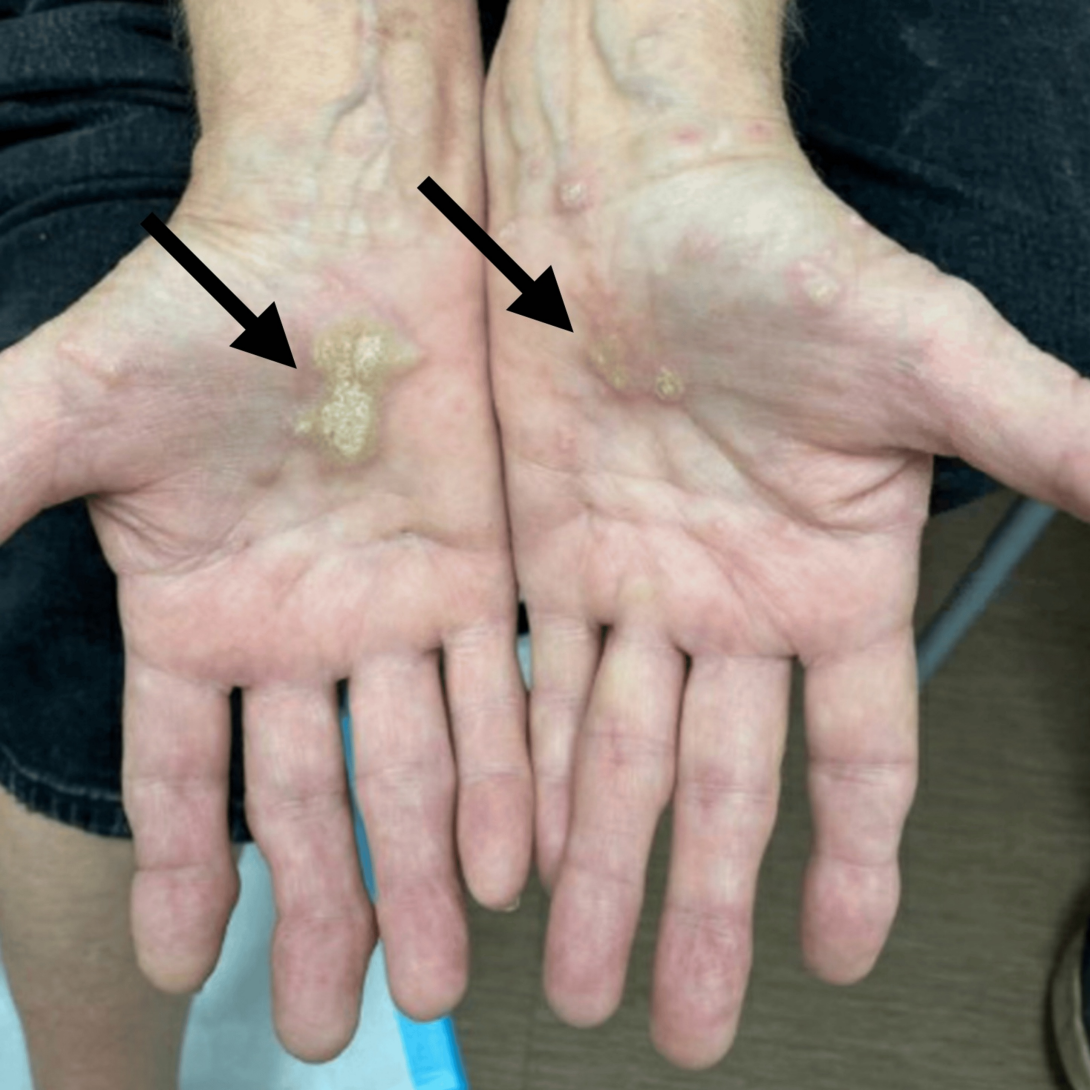
Skin reaction of the palms and wrists that developed following initiation of pembrolizumab for treatment of metastatic glomus tumor

**Figure 7:**
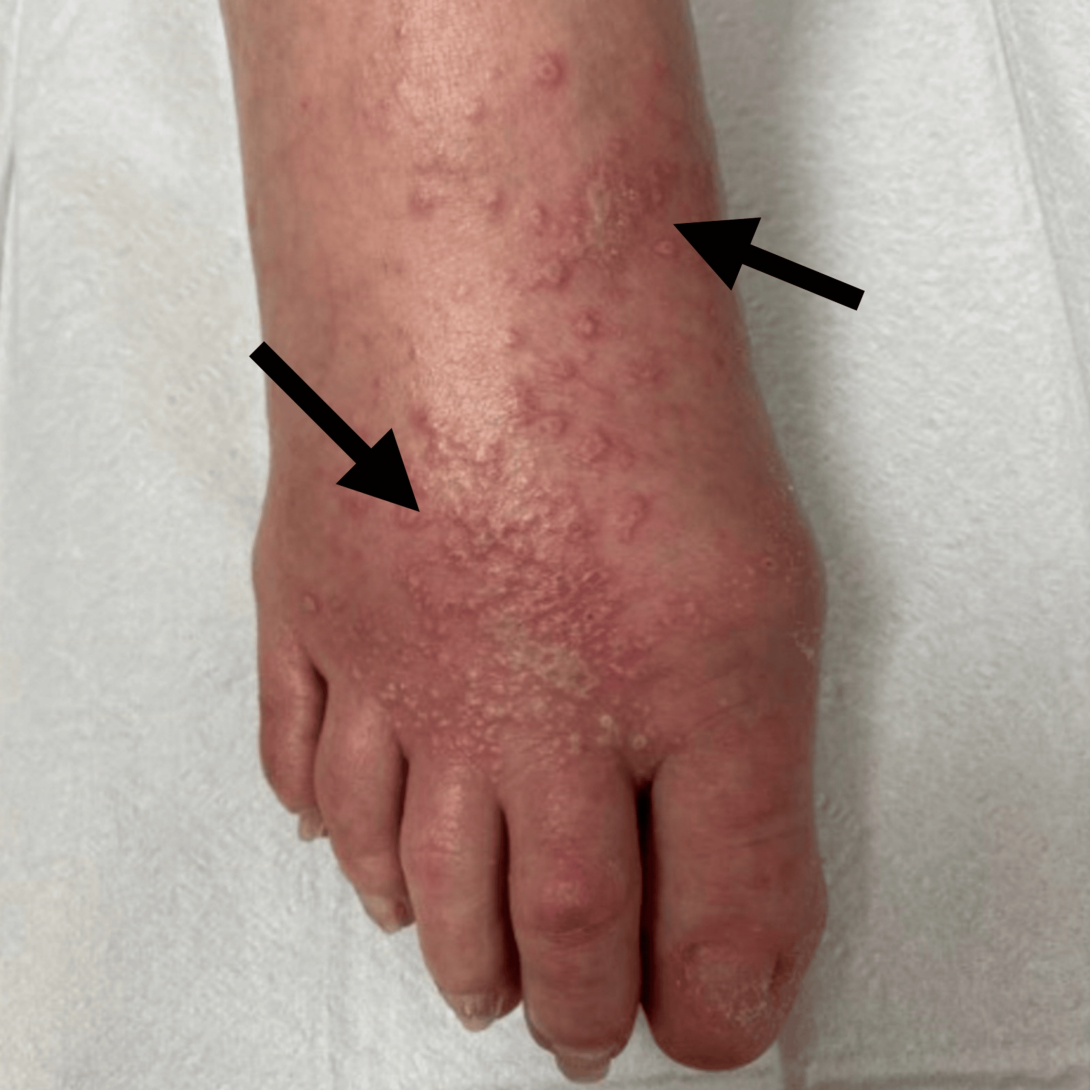
Skin reaction of the dorsal aspect of both feet that developed following initiation of pembrolizumab for treatment of metastatic glomus tumor

**Figure 8:**
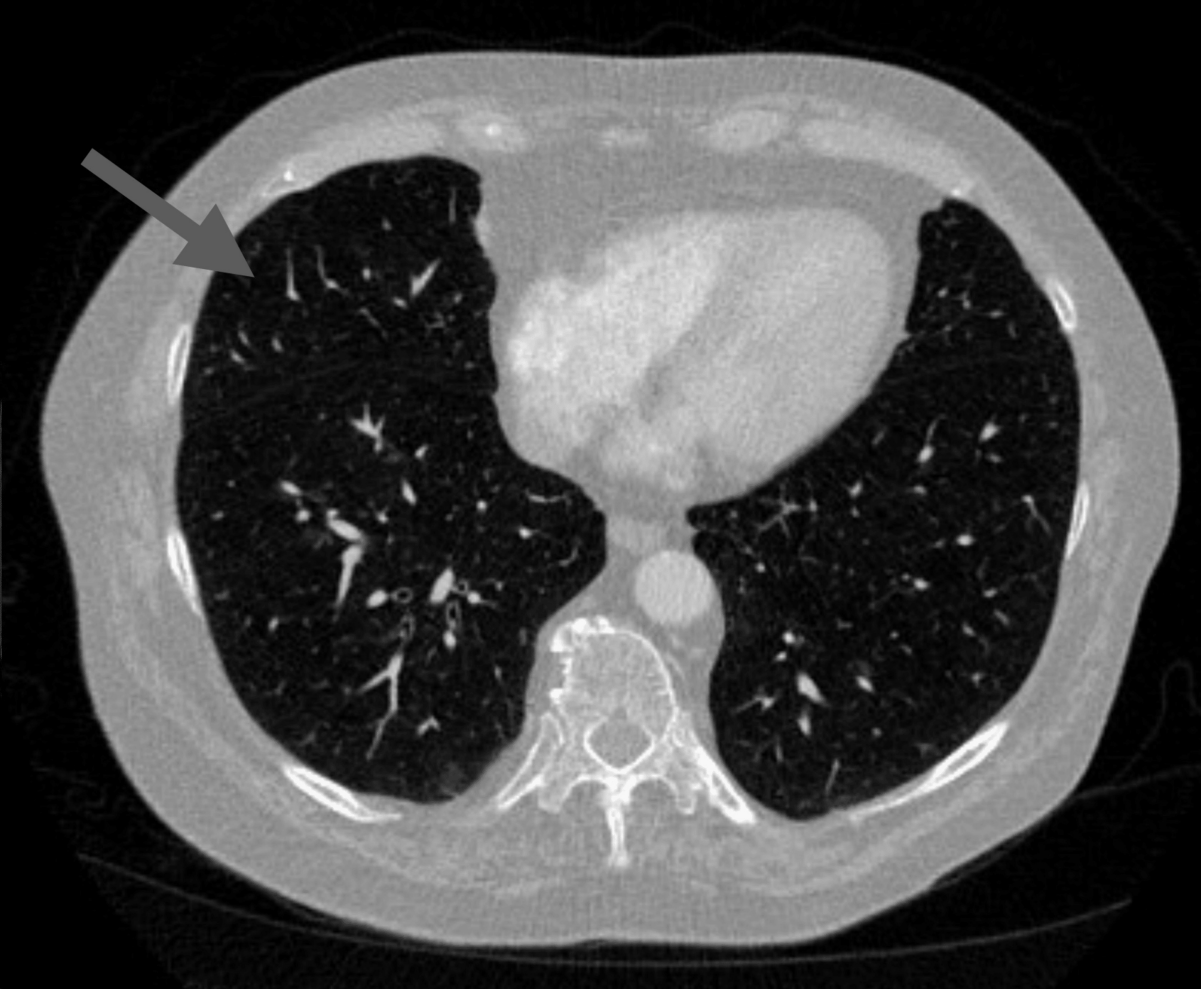
Continued resolution of right middle lobe lung nodules (previously seen in the area indicated by an arrow) following seven months of treatment with pembrolizumab for metastatic glomus tumor

A 67-year-old female was referred to dermatology by her primary care physician for a friable chest wall mass with adjacent skin-colored, subcutaneous nodules, which had been growing rapidly for the last six months (Figure 1). With concern for a neoplastic process, she underwent a shave biopsy of these lesions, and histopathology confirmed a diagnosis of a malignant glomus tumor (Figure 2). She was referred to oncology and underwent computed tomography (CT) imaging of the chest, abdomen, and pelvis, which demonstrated multiple right-sided lung nodules (Figure 3). CT-guided biopsy of a right middle lobe lung nodule was consistent with metastasis of the patient’s known glomus tumor (Figure 4). After a multidisciplinary tumor board discussion, neoadjuvant radiation was initiated. The patient received 30 grays of radiation in ten fractions over 13 days with noted resolution of the subcutaneous nodularity on the anterior chest wall. Next-generation sequencing and neurotrophic tyrosine receptor kinase testing revealed no evidence of microsatellite instability but demonstrated a high tumor mutational burden (TMB-H) of 26 mutations per million bases. There were no other mutations that predicted tumor response for glomus tumors, although other pathogenic mutations were noted, including mutations in neurofibromin 1, cyclin-dependent kinase inhibitor A/B, and tumor protein p53. Pembrolizumab was initiated due to evidence of disease response for TMB-H solid tumors, which may be responsive to anti-programmed cell death protein 1 inhibitors with or without anti-cytotoxic T-lymphocyte-associated protein 4 inhibitors [8]. Prior to the initiation of pembrolizumab, repeat CT imaging of the chest showed regression of the known tumors in the right lung (Figure 5). Four months into treatment with pembrolizumab, the patient developed a severe rash on her palms (Figure 6) and the dorsal aspects of her feet (Figure 7), which improved with topical steroid therapy. Six months into treatment, she developed an acute kidney injury thought to be related to the pembrolizumab and required a brief hospitalization. Her kidney function recovered without complication, and she was continued on treatment for a total of seven months when CT imaging of the chest showed resolution of the lung nodules (Figure 8). With the history of adverse drug reactions, the decision was made to not continue therapy and monitor off-treatment. Since stopping treatment, there has been no reported recurrence of chest wall lesions or lung nodules.

## Discussion

Malignant glomus tumors are quite rare, with one database review finding less than 50 cases from 2000 to 2020 [9]. Despite their rarity, their potential for high mortality makes each case worthy of analysis [6]. Several aspects of this case are unique, including the location of the primary tumor on the chest wall, the metastases found in the lungs, and the cancer’s apparent response to two less common treatments used for malignant glomus tumors.

Glomus tumors have been reported to occur on and within the chest, with instances in the axillary region and within the pleural cavity previously noted [10,11]. This case is unique for its aggressive nature and exterior lesions that developed. As such, their non-specific appearance would have made glomus tumors lower on a differential prior to biopsy. This fact highlights the difficulty with such cases as these vascular tumors require a high degree of suspicion to appropriately diagnose and, therefore, appropriately treat. Recurrences have been reported following resection [12], and with high mortality seen in malignant cases, glomus tumors should be considered when such lesions are encountered.

This patient’s tumor metastases to the lung are an uncommon finding. Glomus tumors have been reported in the respiratory tract but are usually benign primary tumors rather than metastases [13,14]. Depending on the specific location, respiratory glomus tumors can be very dangerous, sometimes resulting in severe complications such as hemoptysis if not treated [13]. Surgical resection can be a treatment option for respiratory glomus tumors [13], but this was not considered given the multiple tumors identified throughout the lungs in this patient. In her case, the pulmonary metastases were never reported to be symptomatic.

The treatments used for this patient were also unique and warrant analysis, given their apparent efficacy. Given metastatic spread to multiple locations, the gold standard of surgical resection was never an option for this patient. Previous cases also remark on the possibility of glomus tumors seeding if surgery is attempted [12]. As discussed above, radiation therapy to the primary site seemed like an appropriate choice for initial treatment and, based on CT imaging, appeared to be effective with both the external chest wall lesion and the lung metastases. The addition of pembrolizumab was also warranted, given the immunophenotype with no microsatellite instability, but its use in glomus tumors is not well studied, with few clinical trials available from which to draw conclusions [8]. Unfortunately, the full effectiveness of radiotherapy could not be determined with this patient as the metastases in the right lung were likely not in the radiation therapy field that was used to treat the chest wall lesions. Since there was a noted regression on CT imaging prior to the initiation of immunotherapy, this implies the possibility of spontaneous regression of this patient’s disease. Additionally, the patient was not continued on pembrolizumab due to multiple adverse drug events. This immunotherapy is well known for having a wide variety of adverse effects, and the skin reactions seen here have been previously reported [15]. Despite this, the patient did achieve remission based on the resolution of the chest wall nodularity and the lung metastases, as seen on imaging. This indicates that the selected treatment course, either partially or in its entirety, was effective against a cancer known to be very aggressive with high mortality rates.

When considering the limitations of this case, it is important to note the ambiguity of treatment benefit. Specifically, it is unclear to what extent the radiotherapy and the immunotherapy contributed to treating the disease versus the possibility of spontaneous regression. There was clear evidence of disease regression on imaging prior to immunotherapy, and the patient continues to be in remission, so this uncertainty continues to persist. An analysis of similar cases in the future shows that trial immunotherapy would be necessary to explore this aspect further. Additionally, this case would require long-term follow-up given the unclear nature of such glomus tumors and the unknown risk of recurrence following treatment.

## Conclusions

Metastatic glomus tumors can be incredibly challenging cancers to diagnose and treat, with few cases from which to draw conclusions. Despite high mortality rates, they are poorly understood in terms of their pathogenesis and treatment. Further research into this tumor type is necessary to create the standard of care. New cancer treatments, such as immunotherapies, provide potential options to help manage these cases and should be considered, especially when surgery is not possible. These rare tumors require both innovative management and a high level of clinical suspicion so that patients get effective care in a timely manner.
